# Superficial Spreading Cervical Squamous Cell Carcinoma Manifesting as Intrauterine Mural Nodules on Magnetic Resonance Imaging

**DOI:** 10.7759/cureus.63808

**Published:** 2024-07-04

**Authors:** Hiroki Takahashi, Risa Ide, Yuri Narusawa, Toshitaka Maejima, Hideyoshi Matsumura

**Affiliations:** 1 Obstetrics and Gynecology, Shinshu Ueda Medical Center, Ueda, JPN; 2 Pathology and Laboratory Medicine, Shinshu Ueda Medical Center, Ueda, JPN

**Keywords:** mural nodules, endometrium, diffusion-weighted imaging, magnetic resonance imaging, superficial spreading, cervical squamous cell carcinoma

## Abstract

Superficially spreading cervical squamous cell carcinoma (SCC) is the superficial extension of SCC of the cervix into the uterine lumen, replacing the endometrium. Here, we report a case of superficially spreading cervical SCC manifesting as intrauterine mural nodules with restricted diffusion on magnetic resonance imaging (MRI). A 76-year-old woman with a history of conization presented with a pelvic mass. MRI revealed a large cystic lesion with mural nodules and wall thickening. The nodular lesions and thickened walls showed high signal intensity on diffusion-weighted imaging (DWI) and low signal intensity on apparent diffusion coefficient (ADC) maps. We performed a laparotomy for diagnosis and treatment and suspected that the tumor was of uterine origin. Hysterectomy and bilateral adnexectomy were performed. Histopathological examination revealed superficial spreading of the cervical SCC. Superficially spreading cervical SCC can manifest as intrauterine mural nodules on MRI. DWI is useful for delineating this disease. If mural nodules or endometrial thickening with restricted diffusion are found in the uterine lumen, clinicians should consider the possibility of the superficial spread of cervical SCC.

## Introduction

Cervical cancer is one of the most common malignancies in women, and squamous cell carcinoma (SCC) accounts for 70-80% of cases [1]. Superficial spreading is a rare form of cervical SCC that extends superficially to the inner surface of the uterus with endometrial replacement [2]. Reports on the magnetic resonance imaging (MRI) findings for this form of SCC are limited [3-8]. There are no reports of diffusion-weighted imaging (DWI) in the English literature. Therefore, the characteristics and usefulness of the MRI findings remain unclear. Here, we report a case of superficially spreading cervical SCC that manifested as intrauterine mural nodules on MRI. DWI is useful for delineating lesions.

## Case presentation

**Figure 1:**
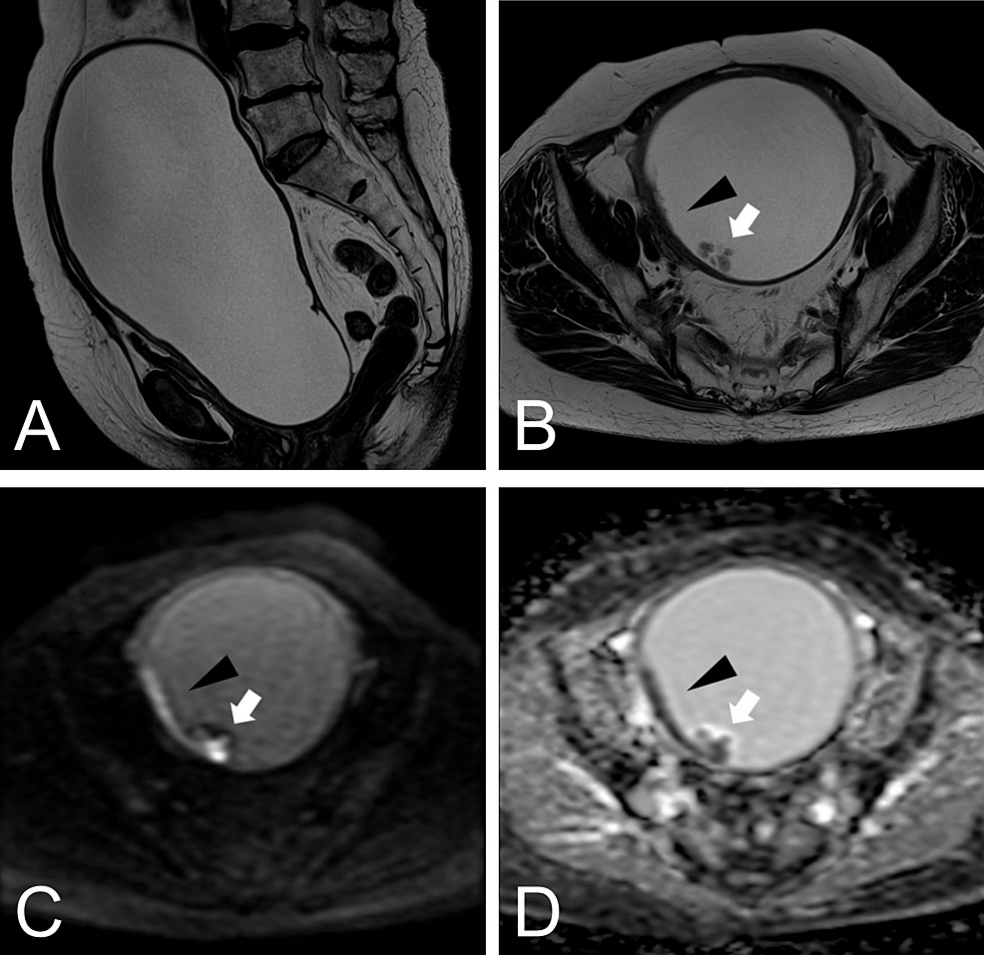
MRI findings (A) Sagittal, T2-weighted MRI section showed an unilocular cystic lesion. (B) Axial T2-weighted MRI. (C) DWI and (D) ADC maps showed a thickened wall (black arrowhead) and mural nodule (white arrow). MRI, magnetic resonance imaging; DWI, diffusion-weighted imaging; ADC, apparent diffusion coefficient

A 76-year-old Japanese woman presented to our hospital with a chief complaint of a lower abdominal mass for the past three months. Ten years ago, the patient had undergone cervical conization and was diagnosed with cervical intraepithelial neoplasia 3 with positive surgical margins. On physical examination, a mass was palpated on the abdomen. The cervix was indistinct and difficult to expand. The cervical and endometrial cytology results were negative for atypical cells. Transvaginal and transabdominal ultrasound failed to identify the uterus and left ovary and showed a large cystic lesion. MRI showed a 200 mm unilocular cystic lesion extending from the pelvis to the umbilical level (Figure 1A). The wall of the cystic lesion was partially thickened. Mural nodules were observed within the cystic lesion (Figure 1B). The thickened and mural nodular areas were hyperintense on DWI and hypointense on the apparent diffusion coefficient (ADC) maps (Figures 1C, 1D).

**Figure 2:**
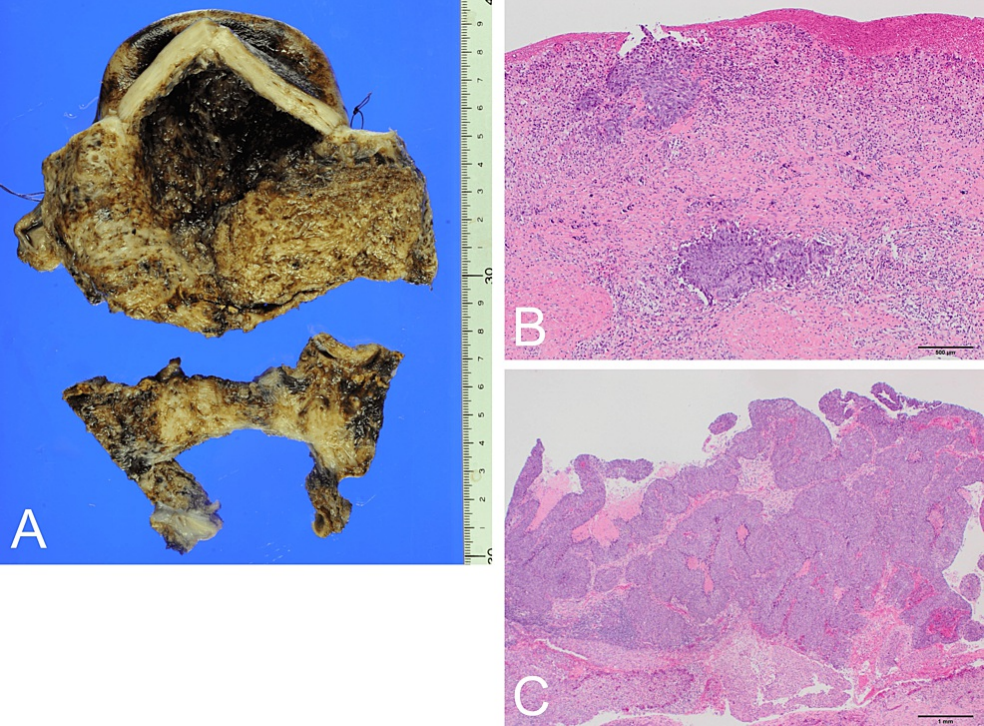
Histopathological findings (A) Macroscopic findings of the uterus. The uterine lumen was dilated, and the wall was slightly thinned. The endometrial surface was generally rough. The cervix was separated from the body by surgical manipulation. (B) Histopathological findings of the cervical mucosa showed atypical epithelial cells in the superficial layer, infiltrating the stroma. (C) Histopathological findings of the endometrium in the uterus showed the outward proliferation of atypical epithelial cells.

Laparotomy was performed for diagnosis and treatment. Intraoperative examination revealed that the uterus enlarged to the size of an adult head. No apparent abnormalities in the serosal surface or adnexa were observed. We punctured the uterine body and aspirated 1300 mL of brown serous fluid. We suspected that the tumor was of uterine origin and performed a hysterectomy and bilateral adnexectomy (Figure 2A). Histopathological findings showed that atypical epithelial cells had formed foci and infiltrated the stroma of the uterine cervix (Figure 2B). Most of the endometrial epithelium detached from the endometrium. Intra-epithelial carcinoma components were also observed in some areas. The outward proliferation almost coincided with the site of the mural nodule on MRI (Figure 2C). Immunohistochemistry revealed that the atypical epithelial cells were positive for p16 and p63. No malignancy was observed bilaterally in the adnexa. We diagnosed cervical SCC with superficial spread to the uterus based on a history of conization, cervical stromal invasion, and p16 positivity. Subsequently, computed tomography (CT) showed lung metastasis, and the patient was scheduled for chemotherapy for stage IVB cervical cancer (the 2018 International Federation of Gynecology and Obstetrics staging system).

## Discussion

This case report highlights two important clinical findings. Superficially spreading cervical SCC can manifest as intrauterine mural nodules on MRI. DWI is useful for delineating this disease.

First, a superficially spreading cervical SCC can manifest as intrauterine mural nodules on MRI. There have been six previous reports of MRI findings of superficially spreading cervical SCC (Table 1) [3-8]. Cervical mass lesions, luminal fluid retention, and endometrial thickening have been reported previously. The Japanese literature also showed irregular and intermittent raised lesions in the endometrium of the uterine body [3]. However, intrauterine mural nodules have not been reported in the English literature. In this patient, a stratified area of atypical cells was histologically found to almost coincide with a mural nodule on MRI. The presence of intrauterine mural nodules may be a novel indicator of superficially spreading cervical SCC. Cervical stenosis is a late complication of conization that results in false-negative cervical cytology [2,9]. It is common for MRI to fail to detect lesions in early-stage cervical cancer because of the small tumor volume [10]. In previous reports of six cases with MRI findings, mass lesions were found in the cervix in four cases [3-6]. Cervical cytology was negative, and MRI showed no cervical abnormalities. Intrauterine mural nodules and wall thickening may be useful for diagnosing and detecting recurrence in these cases.

**Table 1:** Reported cases of MRI findings of superficially spreading cervical SCC MRI, magnetic resonance imaging; SCC, squamous cell carcinoma; DWI, diffusion-weighted imaging; ADC, apparent diffusion coefficient; NA, not available

Reference	Clinical presentation	Mural nodules	Irregular or thickened endometrium	Intrauterine fluid retention	Cervical tumor	DWI/ADC	Contrast-enhanced MRI
Narui et al. [3], 2022	Abdominal mass, abdominal pain	Multiple	+	+	+	High/low	NA
Adler et al. [4], 2007	Dysuria, urinary incontinence, pelvic mass	-	+	+	+	NA	+
Bagde et al. [5], 2021	Discharge	-	+	+	+	NA	NA
Dokić et al. [6], 2022	Bleeding	-	-	-	+	NA	+
Shu et al. [7], 2022	No symptoms	-	-	+	-	NA	-
Mannan et al. [8], 2022	Vaginal discharge, bleeding	-	-	+	-	NA	NA
Current study	Abdominal mass	Multiple	+	+	-	High/low	NA

Second, DWI is useful for delineating superficially spreading cervical SCC. DWI is particularly useful for the evaluation of cervical cancers [11]. It can be used to differentiate benign and malignant uterine lesions [11]. Cervical cancer shows high signal intensity on DWI and low signal intensity on ADC maps, reflecting a high cell density [11]. DWI in superficial spreading cervical SCC has only been reported in the Japanese literature [3] and not in the English literature. In this patient, the nodular lesion and thickened wall showed high signal intensity on DWI and low signal intensity on the apparent ADC map. This finding suggests a superficial extension of cervical cancer to the uterine body. Similar to other gynecological tumors, DWI and ADC maps are useful for the assessment of superficially spreading cervical SCC.

Superficially spreading cervical SCC can manifest as intrauterine mural nodules on MRI. DWI is useful for delineating this disease. If mural nodules or endometrial thickening with restricted diffusion are found in the uterine lumen, clinicians should consider the possibility of the superficial spread of cervical SCC, especially in patients with a history of conization or suspected cervical cancer. MRI findings in superficial spreading cervical SCC are limited. The characteristics and usefulness of MRI findings, including those of DWI and contrast-enhanced MRI, should be further explored. Whether an MRI should be performed to detect the superficial spread of cervical SCC after conization or early-stage cervical cancer should also be investigated.

## Conclusions

Superficially spreading cervical SCC can manifest as intrauterine mural nodules on MRI. DWI is useful for delineating this disease. If mural nodules or endometrial thickening with restricted diffusion are found in the uterine lumen, clinicians should consider the possibility of the superficial spread of cervical SCC.
